# Draft Genome Sequences of Plant-Associated Bacillus Strains Isolated from the Qinghai-Tibetan Plateau

**DOI:** 10.1128/genomeA.00375-18

**Published:** 2018-05-10

**Authors:** Huijun Wu, Rainer Borriss, Pengqi Xue, Fang Liu, Junqing Qiao, Andy Schneider, Peter Lasch, Xuewen Gao

**Affiliations:** a Department of Plant Pathology, College of Plant Protection, Nanjing Agriculture University, Nanjing, China; b Nordreet UG, Greifswald, Germany; c Institute of Marine Biotechnology e.V. (IMaB), Greifswald, Germany; d Proteomics and Spectroscopy Unit (ZBS6) at the Centre for Biological Threats and Special Pathogens, Robert Koch-Institut, Berlin, Germany

## Abstract

Here, we report the draft genome sequences of 45 plant-associated Bacillus strains isolated from the Qinghai-Tibetan plateau. According to their genome sequences, 28 isolates were assigned to 10 Bacillus species. Seventeen strains could not be assigned and are subjects of further research.

## GENOME ANNOUNCEMENT

Bacillus strains isolated from samples taken from different sites of the Qinghai-Tibetan plateau, known as the Third Pole of the world (1, 2), were found to grow significantly between 4°C and 12°C (H. Wu, R. Borriss, P. Xue, F. Liu, and X. Gao, unpublished data), to enhance plant growth, and to suppress plant pathogens (3–5). As a first step to characterize these strains more deeply, 45 of the isolates were genome sequenced and their taxonomy was determined.

Colonies of a fresh culture grown on LB agar plates were selected. Genomic DNA was extracted using the QIAamp DNA minikit (Qiagen, Hilden, Germany), and the sequencing was done in 300-nucleotide (nt) paired-end mode on an Illumina MiSeq version 3 sequencing platform at LGC Genomics (Berlin, Germany). Reads were trimmed and assembled *de novo* using the A5 pipeline (6). Genome coverage of the obtained scaffolds was 45× on average. Scaffolds were submitted to GenBank for gene annotation, which was implemented using the NCBI Prokaryotic Genome Annotation Pipeline (PGAP) (7). The genome-to-genome-distance calculator (GGDC) version 2.1 provided by DSMZ (http://ggdc.dsmz.de) was used for genome-based species delineation. Formula 2, which is especially appropriate to analyze draft genomes, was used (Meier-Kolthoff et al., 2013 [8]). In addition, JSpecies WS (http://jspecies.ribohost.com/jspeciesws/) was used to determine the average nucleotide identity based on BLAST+ (ANIb) by pairwise genome comparisons (9). The recommended species cutoff was defined as 96%.

According to their draft genome sequences, we have assigned 28 of the isolates as representatives of Bacillus wiedmannii (GenBank accession numbers PVRQ00000000 to PVRU00000000, and PYWP00000000), B. atrophaeus (PVQM00000000 to PVQO00000000, PVWA00000000, and PVWB00000000), B. pumilus (PVQT00000000 to PVQX00000000), B. halotolerans (PVWC00000000, PVQP00000000, and PVQQ00000000), B. subtilis (PVRJ00000000 and PVRK00000000), B. thuringiensis (PVRL00000000 and PVRM00000000), B. velezensis (PVRO00000000 and PVRP00000000), B. paralicheniformis (PVQR00000000), B. safensis (PVQS00000000), and B. toyonensis (PVRN00000000). Seventeen strains could not be assigned down to the species level due to their estimated GGDC (<70%) and ANIb (<96%) values. Most of the strains (15 isolates) are related to B. pumilus (PVQY00000000, PVQZ00000000, PVRA00000000 to PVRI00000000, PVQK00000000, PVQL00000000, PVWX00000000, and PVWY00000000). The genome sequence of strain RJGP41 (PVQJ00000000) is distantly related to B. simplex, while strain LLTC93 (PVME00000000) resembles the type strain of B. xiamenensis, HYC-10. Further research is in progress in order to clarify the taxonomic position of these cold-adapted strains.

### Accession number(s).

These whole-genome shotgun projects have been deposited at DDBJ/ENA/GenBank under the accession numbers PVME00000000, PVQJ00000000, PVQK00000000, PVQL00000000, PVQM00000000, PVQN00000000, PVQO00000000, PVQP00000000, PVQQ00000000, PVQR00000000, PVQS00000000, PVQT00000000, PVQU00000000, PVQV00000000, PVQW00000000, PVQX00000000, PVQY00000000, PVQZ00000000, PVRA00000000, PVRB00000000, PVRC00000000, PVRD00000000, PVRE00000000, PVRF00000000, PVRG00000000, PVRH00000000, PVRI00000000, PVRJ00000000, PVRK00000000, PVRL00000000, PVRM00000000, PVRN00000000, PVRO00000000, PVRP00000000, PVRQ00000000, PVRR00000000, PVRS00000000, PVRT00000000, PVRU00000000, PVWA00000000, PVWB00000000, PVWC00000000, PVWX00000000, PVWY00000000, and PYWP00000000. The versions described in this paper are the first versions.
